# Paroxetine in the treatment of premature ejaculation: a systematic review and meta-analysis

**DOI:** 10.1186/s12894-018-0431-7

**Published:** 2019-01-03

**Authors:** Dong Zhang, Yue Cheng, Kerong Wu, Qi Ma, Junhui Jiang, Zejun Yan

**Affiliations:** Department of Urology & Nephrology, Ningbo First Hospital, The affiliated hospital of ZheJiang University, 59, Liuting Street, Ningbo, Zhejiang, China

**Keywords:** Premature ejaculation, Paroxetine, Meta-analysis, Systematic review

## Abstract

**Background:**

Paroxetine is one of the selective serotonin reuptake inhibitors (SSRIs) used in the treatment of premature ejaculation (PE). However, this use is not approved in many countries. The purpose of this systematic review and meta-analysis is to review the efficacy and safety of paroxetine for PE patients.

**Methods:**

We searched relevant randomized, controlled trials through May 2018, using PubMed, Embase and Cochrane Central Register. The main endpoint included intra-vaginal ejaculatory latency time (IELT) and side effects in the treatment of PE. Cochrane Collaboration’s Revman software, version 5.3, was used for statistical analysis.

**Results:**

Out of 493 unique articles, a total of 19 randomized, controlled trials (RCTs) were reviewed. Quite a few RCTs were considered to have unclear risk of bias because of limited information. Pooled outcomes suggested that paroxetine was more effective than placebo, fluoxetine and escitalopram at increasing IELT (all *p* < 0.05). However, there existed a high level of heterogeneity in the paroxetine vs. fluoxetine groups and the paroxetine vs. placebo groups. Comparing paroxetine with tramadol, sertraline, phosphodiesterase 5 inhibitors (PDE5Is), local lidocaine gel, behaviour therapy or dapoxetine, we found that the increase in IELT was not statistically significant between groups. Paroxetine combined with tadalafil or behaviour therapy was more efficacious than paroxetine alone (all *p* < 0.05). Although the side effects in the combination group were more common than in the paroxetine alone group, the most common adverse events, such as nausea, muscle soreness, palpitation and flushing, were mild and tolerable. The main limitations of this systematic review and meta-analysis were the different definitions of PE and short follow-up times.

**Conclusions:**

According to this systematic review and meta-analysis, paroxetine provided better efficacy than placebo, fluoxetine and escitalopram in the treatment of PE, with well-tolerated side effects. The combination group had better efficacy than the paroxetine alone group.

**Trial registration:**

This review was reported in agreement with the PRISMA statement and was registered on PROSPERO 2018CRD42018097014.

## Background

Premature ejaculation (PE) is recognized as one of the most common diseases of sexual dysfunction, affecting approximately 20–30% of men [1]. It has been proved that PE can influence the quality of intercourse, resulting in distress and anxiety and even impacting the relationships between partners [2]. While the aetiology of PE remains controversial,it is increasingly becoming recognized that [3–5] psychological problems, somatic disorders and/or neurobiological disturbances, and polymorphisms of the serotonin transporter or its promoters frequently co-occur in the same individual. However, other potential aetiologic factors [6–10], including depression,erectile dysfunction,metabolic syndrome,chronic prostatitis and thyroid dysfunction, have been definitely established as causative in PE.

According to the new International Society for Sexual Medicine (ISSM) guidelines [11], PE is defined as “ejaculation that always occurs after less than 1 minute of vaginal penetration from the first sexual experience (lifelong PE), or a clinically significant and bothersome reduction in latency time, often ≤3 minutes (acquired PE),and the inability to delay ejaculation on all or nearly all vaginal penetrations,and negative personal consequences, such as distress, frustration and/or the avoidance of sexual intimacy.” Therefore, we use intravaginal ejaculatory latency time (IELT) to evaluate the endpoint of PE.

Over the past few decades, many feasible therapies have been explored by andrologists, such as selective serotonin reuptake inhibitors (SSRIs), topical anaesthetics, tricyclic antidepressants, PDE5Is, α-receptor blockers and surgery. Although, many of them have been reported to be useful for PE with well-tolerated side effects, none of them has been approved by the FDA (Food and Drug Administration) except for dapoxetine. Paroxetine is one of the SSRIs, which increase the amount of 5-hydroxytryptamine (5-HT) in postsynaptic membrane receptors and thus delay ejaculation. Although it is not approved by the FDA, it has the advantage of lower dropouts and cost, with almost identical effects to dapoxetine [12, 13]. Recently, there have been numerous high-quality randomized, controlled trials (RCTs) comparing paroxetine with other therapeutic options for the treatment of PE. To our knowledge, this study is the first meta-analysis to report on the efficacy and safety of paroxetine in the treatment of PE. The purpose of this systematic review and meta-analysis is to review the efficacy and safety of paroxetine for PE patients.

## Methods

### Inclusion and exclusion criteria

The study inclusion criteria were diagnosis of PE but not erectile dysfunction, a stable relationship with the same sexual partner, and RCTs comparing paroxetine with other medical therapies for PE.

The study exclusion criteria were diabetes, hepatic or renal impairments, urogenital diseases, patients with ejaculation dysfunction, quasi-randomized trials, non-randomized trials, observational studies, case reports, and abstracts and letters.

### Literature search and data sources

We used subject terms (MeSH) including “premature ejaculation” and “paroxetine” with their free words to search for relevant clinical trials through May 2018 in PubMed, Embase and Cochrane Central Register. The complete search used for PubMed was (premature ejaculation [MeSH terms] OR premature ejaculation [Text word]) AND (paroxetine [MeSH terms] OR paroxetine [Text word]). The primary research process was to find the whole articles that were relevant to paroxetine and other drugs for PE. Then, the eligible RCT articles were collected based on our criteria. All of the processes were independently completed by two authors. Consensus was reached by discussion if there was any disagreement. We tried our best to contact the corresponding authors if data were missing.

### Data extraction

One reviewer read the articles and compiled notes of the authors, date of publication, drugs and dosage, number of participants, clinical effects, side effects and PE types. All numerical data were then checked by the other reviewer.

### Quality assessment and statistical analysis

According to the Cochrane risk of bias tool [14] (including bias of selection, performance, detection, attrition, reporting, and other), we could define each item as low risk or unclear or high risk, finally devising a risk of bias summary graph. Two reviewers complete the quality assessment of each study. We used Review Manager software, version 5.3 (Cochrane Collaboration, Oxford, United Kingdom), to analyse dichotomous and continuous data on side effects and IELT, respectively. A fixed or the random effect model was applied for meta-analysis according to the value of heterogeneity, which was assessed by the Mantel-Haenszel chi-square test and I^2^ statistic. If the *p* value was< 0.1 and I^2^ > 50%, it was suggested that the heterogeneity was unacceptable, and sensitivity analysis should be performed. We used the mean difference (MD) to compare the IELT and relative risk (RR) to compare side effects between the different groups. Funnel plots were used to assess the publication bias if more than 10 RCTs were included in a comparison. A *p* value less than 0.05 was considered to indicate statistical significance. The confidence interval was established at 95%.

## Results

### Description of studies

#### Search results and reporting quality

**Fig. 1:**
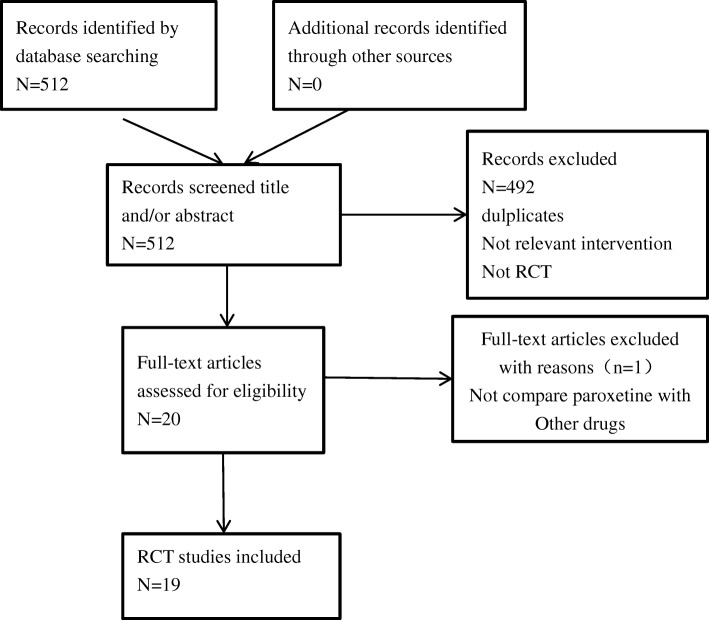
Flow diagram of the study selection process. RCT = randomized controlled trail

After searching the 3 databases, a total of 512 relevant articles were retrieved. According to the titles and abstracts of publications, 492 records were excluded because of repeats, irrelevance or not being RCTs. The remaining 20 full-text articles were uploaded and assessed for eligibility, and 19 of 20 met our criteria. Thus, 19 RCTs were included for systematic review and meta-analysis. Table 1 shows their characteristics, and Fig. 1 summarizes the inclusion process. Comparators included placebo, dapoxetine, tramadol, sertraline, PDE5Is, fluoxetine, behaviour therapy, local lidocaine gel, duloxetine, escitalopram, and combined therapy. The majority of the included RCTs were 4–12 weeks in duration. Only Wang’s and Moudi’s trials lasted for 6 months [15, 16]. All of the articles reported similar outcomes involving IELT. Other diffused distribution outcomes included sexual satisfaction score, side effects, premature ejaculation diagnostic tool (PEDT), International Index of Erectile Function (IIEF), premature ejaculation profile (PEP), Arabic Index of Premature Ejaculation (AIPE), libido and frequency of intercourse, which rendered pooled meta-analysis difficult. The only method that we could use was to depict these outcomes. If study data were missing, we attempted to contact the corresponding authors.

**Table 1:** Characteristics of included stuides

RCT, year, dose, duration	Treatment (numbers)	Outcomes	Adverse events	IELT (SD)	PE definition
Gammel et al. [20] 2013 20 mg 4 weeks Sunay et al. [21] 2011 20 mg 4 weeks Safarinejad et al. [22] 2006 20 mg 12 weeks	on-demand tramadol 50 mg/d (29), sildenafil 50 mg/d (30), paroxetine 20 mg/d (28), local lidocaine gel (30), placebo (27) daily paroxetine 20 mg/d (30), acupuncture (30), placebo (30), daily dapoxetine 60 mg/d (104), paroxetine 20 mg/d (105), placebo (100), daily dapoxetine 60 mg/d (104), paroxetine 20 mg/d (105), placebo (100)	IELT, sexual satisfaction scores IELT, PEDT IELT, IIEF, weekly intercourse episodes IELT, IIEF, weekly intercourse episodes	all adverse events were tolerable no side effects were observed well tolerated	5.85 (1.98), 3.8 (1.15), 3.1 (1.08), 2.97 (1.85), 1.35 (0.54) 1.17, 1.17, 0.42 2.98, 6.17, 0.92	IELT < 2 min in > 70% of sexual intercourse episodes IELTs of < 2 min in > 70% of coital attempts IELT < 2 min that occurred in > 90% of episodes of sexual inter course
Gong et al. [18] 2011 20 mg 4 weeks Ozcan et al. [33] 2015 20 mg 1 month	daily paroxetine 20 mg/d (40), placebo (40) daily duloxetine 40 mg/d(40), daily paroxetine 20 mg/d (40)	IELT, sexual satisfaction scores IELT, IIEF, PEP	well tolerated well tolerated	5.75 (1.24), 1.06 (0.28) 2.09 (0.12), 2.14 (0.15)	DSM-IV-TR lifelong PE
Abdel-Hamid et al. [24] 2001 20 mg 4 weeks	on-demand clomipramine 25 mg, on-demand sertraline 50 mg, on-demand paroxetine 20 mg, on-demand sildenafil 50 mg and the pause-squeeze technique	IELT, sexual satisfaction scores	mild to moderate	4, 3, 4, 15, 3	IELT ≤2 min
Abu et al. [23] 2018 30 mg 6 weeks	on-demand sildenafil 50 mg/d combined with dapoxetine 50 mg/d (30), sildenafil 50 mg/d (30), paroxetine 30 mg/d (30), dapoxetine 30 mg/d (30), placebo (30)	IELT, PEDT, sexual satisfaction	well tolerated	4.43, 2.93, 2.90, 2.86, 0.69	IELT ≤1 min
Otunctemur et al. [27] 2014 20 mg 4 weeks	daily fluoxetine 20 mg/d (20), sertraline 50 mg/d (20), paroxetine 20 mg/d (20), healthy control (40)	IELT	not mentioned	2.5 (0.69), 3.09 (1.15), 3.70 (0.86)	IELT < 1 min
KIrecci et al. [29] 2014 20 mg 4 weeks a	daily sertraline 50 mg/d (8), paroxetine 20 mg/d (8), healthy control (11)	IELT, IIEF	not mentioned	1.89 (0.51), 1.92 (0.49)	IELT < 1 min, primary PE
Kirecci et al. [28] 2014 20 mg 4 weeks b	daily sertraline 50 mg/d (20), paroxetine 20 mg/d (20), fluoxetine 20 mg/d (20)	IELT, IIEF	tolerated	1.71 (0.98), 1.78 (0.62), 1.55 (0.64)	lifelong PE (IELT of < 1 min)
Wang et al. [15] 2007 20 mg 6 months	on-demand sildenafil 50 mg/d (59), paroxetine 20 mg/d (649), squeeze technique (38)	IELT, PE grade, intercourse satisfaction score, frequency of intercourse	well tolerated	6.21 (1.86), 4.93 (1.36), 2.62 (0.69)	IELT < 2 min, primary PE
Alghobary et al. [25] 2010 20 mg 6 weeks	on-demand tramadol 50 mg/d (17), daily paroxetine 20 mg/d (18)	IELT, AIPE, libido, erection	well tolerated	3.00,3.84	DSM-IV-TR, lifelong PE
Polat et al. [26] 2015 20 mg 1 month	daily paroxetine 20 mg/d (50), on-demand tadalafil 20 mg (50), on-demand paroxetine 20 mg combined tadalafil 20 mg (50)	IELT, IIEF	well tolerated	1.96 (1.12), 1.84 (0.62), 2.92 (1.00)	lifelong PE
Waldinger et al. [19] 1998 20 mg 6 weeks	placebo (9), fluvoxamine 100 mg/d (10), fluoxetine 20 mg/d (10), paroxetine 20 mg/d (11), sertraline 50 mg/d (11),	IELT	well tolerated	0.48 (0.41), 55 (70), 3.5 (4.18), 7.9 (19.1), 1.95 (1.45)	lifelong PE (IELT of < 1 min)
Shao et al. [32] 2008 20 mg 8 weeks	paroxetine 20 mg/d (40), behaviour therapy (40), paroxetine 10 mg/d combined behaviour therapy (40)	IELT, CIPE-5	well tolerated	4.4 (0.5), 4.2 (0.4), 4.8 (0.5)	not mentioned
Moudi et al. [16] 2016 10 mg 6 months Zhang et al. [30] 2012 20 mg 12 weeks Hamidi-Madani et al. [17] 2018 20 mg 12 weeks Arafa et al. [31] 2007 20 mg 4 weeks	paroxetine 10 mg/d (50), paroxetine 10 mg/d combined tadalafil 10 mg/d (50) behaviour therapy (22), paroxetine 20 mg/d (32), paroxetine 30 mg/d (32), daily sertraline 50 mg/d (28), daily sertraline 100 mg/d (30) on-demand tramadol 50 mg/d (48), paroxetine 20 mg/d (46), placebo (32) daily fluoxetine 20 mg/d (33), escitalopram 10 mg/d (37), paroxetine 20 mg/d (30)	IELT, IIEF IELT, sexual satisfaction IELT, PEP AIPE, IELT	well tolerated well tolerated well tolerated, well tolerated,	4.8 (1), 5.3 (2)—— 2.29 (1.29), 1.52 (1.23), 1.30 (0.92) 2.4 (0.4), 2.5 (0.3), 2.7 (0.2)	lifelong PE (IELT of < 1.5 min) not mentioned lifelong PE (IELT of < 1 min) IELT ≤2 min

*RCT* randomized, controlled trial, *IELT* intravaginal ejaculatory latency time, *PEP* premature ejaculation profile, *IIEF* international index of erectile function, *AIPE* Arabic index of premature ejaculation, *CIPE-5* Chinese index of premature ejaculation, *PEDT* premature ejaculation diagnostic tool, *PE* premature ejaculation, *SD* standard deviation, *DSM-IV* Diagnostic and Statistical Manual of Mental Disorders, fourth edition

**Fig. 2:**
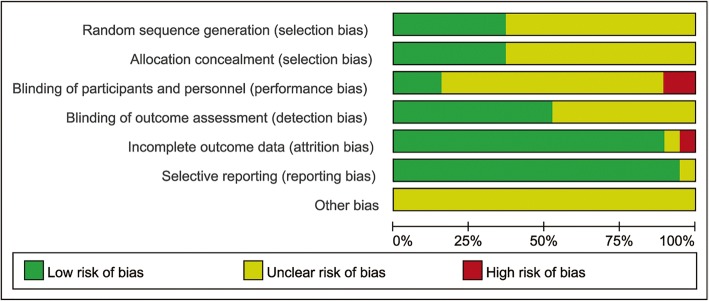
Overall quality assessment for the selected articles

Cochrane’s risk of bias tool was used to assess the quality of the articles on the basis of random sequence generation, allocation concealment, blinding of participants and personnel, blinding of outcomes assessment, incomplete outcome data, selective report and other biases. Then, a risk of bias summary graph was successfully generated, as Fig. 2 shows. We can see that a large number of RCTs were considered to have an unclear risk of bias because of a lack of adequate information from the articles. Gameel’s and Abu’s trails were considered at high risk of performance bias due to single blinding. One RCT was considered at high risk of attrition due to incomplete outcome data: there were 1.67, 18.33 and 36.67% of patients in the sildenafil, paroxetine and squeeze therapy groups, respectively, who withdrew from the trial due to little efficacy or side effects [15].

#### Efficacy

**Fig. 3:**
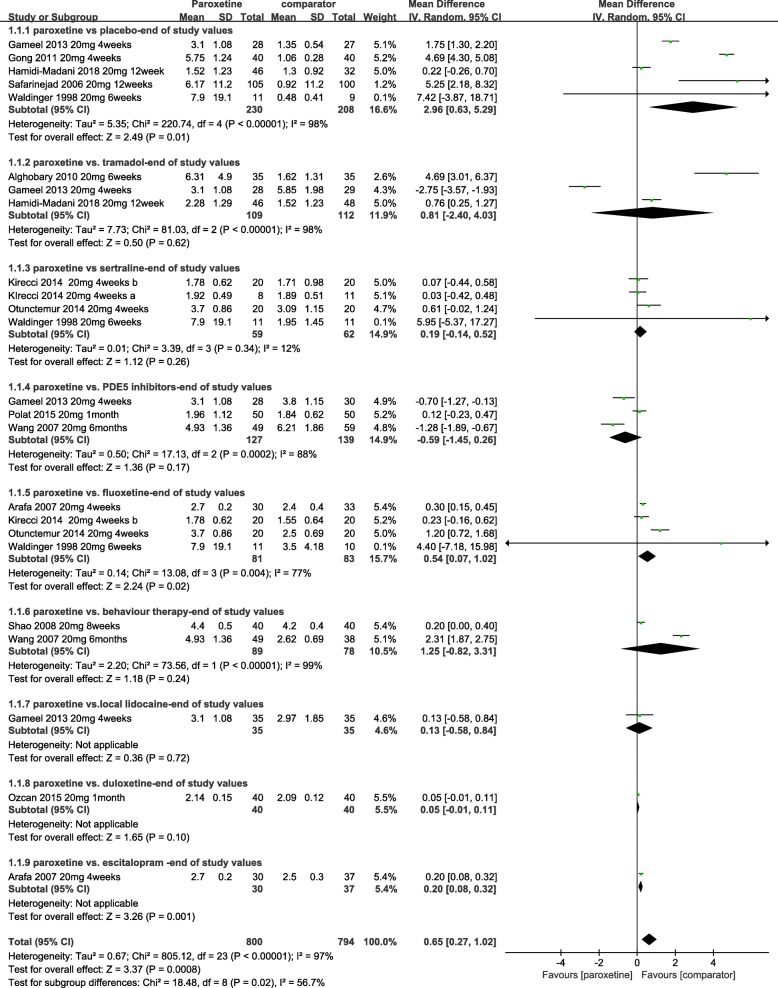
Forest plot of IELT between paroxetine and other drugs. Cl,confidence interval;IELT, intra-vaginal ejaculatory latency time; PDE-5, phosphodiesterase-5 inhibitor; SD, standard deviation

Paroxetine vs. placebo: A total of 7 RCTs met the condition [17–23]. Two RCTs lacked relevant standard deviations [21, 23]. The quality of these articles were described,but they were not included in the meta-analysis. Thus,based on 5 pooled RCTs [17–20, 22], the men treated with paroxetine 20 mg for 4–12 weeks had significantly increased IELT compared with placebo (*p* = 0.01). The MD in IELT was 2.96, in favour of paroxetine [(random effect) 95% confidence interval [Cl], 0.63 to 5.29; *p* = 0.01] (Fig. 3). Meta-analysis of these studies showed a high level of heterogeneity, which might have arisen from the difference in types of PE and treatment periods (Fig. 3). Seven RCTs favoured of paroxetine. One RCT [17] showed PEP changes from the baseline significantly greater than placebo, and two RCTs [18, 20] showed that the change in satisfaction score from baseline was more significant. One RCT reported that paroxetine had a significantly stronger ejaculation-delaying effect than placebo (*p* < 0.05), and it decreased PEDT significantly more than placebo without any side effects [21]. Safarinejad et al. [22] reported that paroxetine had better ability to delay ejaculation than placebo at the end of 12 weeks of treatment. Not only was IELT increased from 31 and 34 to 370 and 55, respectively, with paroxetine (*p* < 0.05) and placebo (*p* > 0.05), but the mean number of coitus episodes and IIEF value improved significantly in the paroxetine group (*p* < 0.05). A similar finding was reported by Abu et al. [23]. The IELT significantly improved from 38.66 to 173.86 in the paroxetine group, while the placebo group showed almost no change. The mean satisfaction score and PEDT in the paroxetine group improved more significant than with placebo. Therefore, paroxetine could have a significantly stronger ejaculation-delaying effect than placebo.

**Fig. 4:**
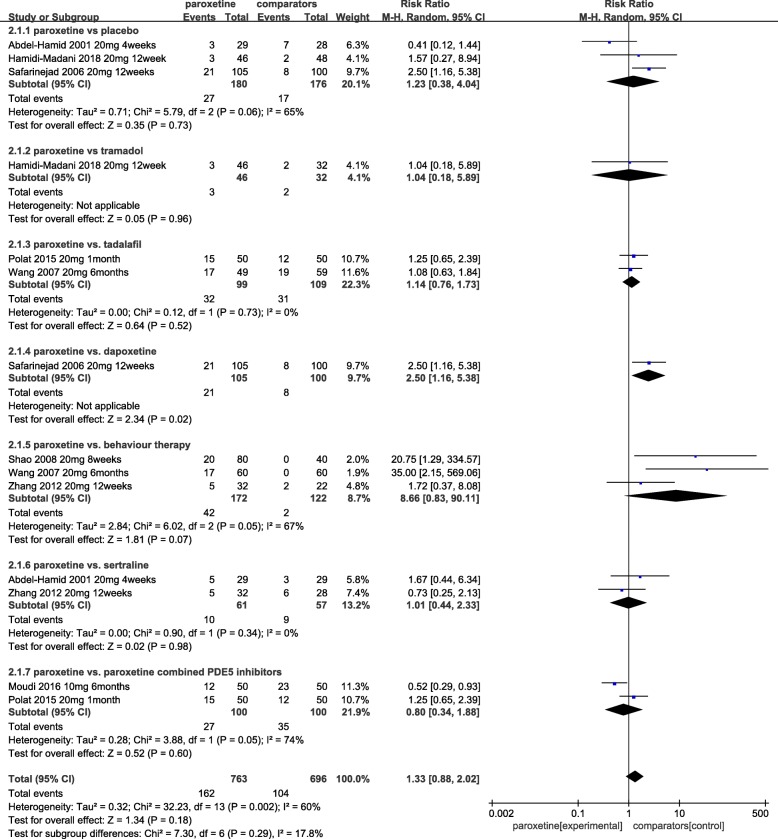
Pooled estimate of side effects of paroxetine vs. other medical therapy

Where reported, side effects related to paroxetine included: headache, fatigue, nausea, dizziness, sleep disturbances, yawning, dry mouth, sweating, and constipation. The pooled relative risk of 3 RCTs [17, 22, 24] was 1.23 [RR (random effect) 95% Cl, 0.38 to 4.04; *p* = 0.73], which indicated no difference between the paroxetine and placebo groups in terms of adverse events (Fig. 4). Our pooled estimate showed significant heterogeneity (I^2^ = 65%), which might have arisen from the treatment period, dosage and inclusion criteria. Four RCTs [18–21] that followed up for 3–10 weeks showed no serious treatment-related side effects detected for paroxetine or placebo.

Paroxetine vs. tramadol: Three RCTs [17, 20, 25] provided evidence that suggested that the difference in IELT was not significant between the two groups [MD, 0.81; 95% [Cl], − 2.40 to 4.03; *p = 0.62*] (Fig. 3). One of 3 [17] reported that on-demand tramadol more significantly improved PEP than paroxetine at 4 weeks. Another RCT reported that there was no difference in terms of sexual satisfaction score after one month of on-demand treatment [20]. In addition, Alghobary et al. [25] compared the efficacy of daily paroxetine and on-demand tramadol, paroxetine and tramadol increased IELT after 6 weeks by 11- and 7-fold, respectively. After 12 weeks, the tramadol group decreased IELT to fivefold, while the paroxetine group increased IELT to 22-fold. The tendency of the Arabic Index of PE (AIPE) was consistent with IELT in the two groups. Paroxetine increased libido significantly more than tramadol at 12 weeks. Therefore, a longer treatment time should be used to explore the efficacy and safety of tramadol and paroxetine.

Side effects related to tramadol were sleep disturbance, dry mouth, vomiting and nausea, dizziness, fatigue, sweating, constipation and headache, which were similar with paroxetine (*p* = 0.96) [RR, 1.04; 95% [Cl] 0.18 to 5.89] (Fig. 4). All of the articles showed that paroxetine and tramadol have well-tolerated side effects [17, 20, 25].

Paroxetine vs. PDE5Is: Five RCTs compared the safety and efficacy of paroxetine with those of tramadol [15, 20, 23, 24, 26]. Because 2 RCTs lacked relevant standard deviations [23, 24],3 other pooled RCTs [15, 20, 26] showed that paroxetine had similar effects to PDE5Is,with between-group difference in IELT of − 0.59 [95% [Cl], − 1.45 to 0.26;*p* = 0.17]. While there was a high level of heterogeneity (I^2^ = 88%) (Fig. 3), which may come from the difference type of PE and treatment period. One RCT [20] showed that the on-demand sildenafil group has better sexual satisfaction scores than the daily paroxetine group. Wang et al. [15] reported that 1.7 and 18.3% of patients withdrew from the study in the sildenafil and paroxetine groups, respectively, after 6 months. One RCT [23] reported that there was no significant difference in PEDT or satisfaction score after 6 weeks of treatment between the two groups.

The relative risk of side effects between two groups pooled from 3 RCTs [24, 26] was 1.14 [RR (random effect) 95% Cl, 0.76 to 1.73; *p* = 0.52], as shown in Fig. 4. According to 5 RCTs, all side effects were well tolerated. One RCT showed that sleep disturbances, dry mouth, nausea, dizziness, fatigue, vomiting, sweating, headache, flushing, hypotension and nasal congestion were reported with paroxetine and sildenafil [20]. One RCT [23] reported that the most adverse effect in the paroxetine group was sleep disturbance, and in the sildenafil group, it was headache.

Paroxetine vs. dapoxetine: The IELT with treatment using paroxetine and dapoxetine increased from 38 and 31 to 37 and 179, respectively, at the end of 12 weeks in one RCT [22]. Sexual satisfaction was also significantly higher with paroxetine than with dapoxetine (*p* = 0.04). According to analysis of variance with multiple comparisons, treatment with paroxetine caused a greater increase in mean weekly intercourse frequency than dapoxetine. Abu et al. [23] reported that paroxetine resulted in higher satisfaction scores and IELT than dapoxetine, although the difference was not statistically significant.

A single RCT [22] showed that there were significantly more side effects in the paroxetine group than in the dapoxetine group [RR, 2.50; 95% Cl, 1.16 to 5.38]. Drug-related side effects with dapoxetine included headache (6.6%), fatigue (10%), nausea (26.6%), dizziness (10%), sleep disturbances (13.3%) and yawing (16.7%) [23]. A forest plot is presented in Fig. 4.

Paroxetine vs. sertraline: Six studies [19, 24, 27–30] investigated IELT and the side effects with paroxetine vs. sertraline. In 4 pooled RCTs [19, 27–29], treatment with paroxetine was more effective than sertraline, but the difference was not statistically significant [MD, 0.19; 95% Cl, − 0.14 to 0.52; *p* = 0.26] (Fig. 3). There was no evidence of statistical heterogeneity between the groups as assessed by the χ^2^ test (χ^2^ = 3.39; I^2^ = 12%; *p = 0.34*) (Fig. 3). One RCT reported that paroxetine had greater efficacy than sertraline [24], while Zhang et al. [30] showed that there was no difference between the two groups in terms of IELT and sexual satisfaction score. The relative risk of side effects between the two groups pooled from 2 RCTs [24, 30] was 1.01 [RR (random effect)95% Cl, 0.44 to 2.33; *p* = 0.98], as shown in Fig. 4. All side effects were tolerable.

Paroxetine vs. fluoxetine: The between-group difference in IELT at 4–6 weeks, based on 4 RCTs [19, 27, 28, 31] comparing daily paroxetine with fluoxetine, was 0.54 in favour of paroxetine [95% Cl, 0.07 to 1.02; *p* = 0.02] (Fig. 3). The AIEF between groups was not significantly different [31]. The drug-related side effects were tolerable.

Paroxetine vs. behaviour therapy: Evidence from two RCTs [15, 32] suggested that the difference in IELT was not significant between the two groups [MD, 1.25; 95% [Cl], − 0.82 to 3.31;*p* = 0.24] (Fig. 3). One of 2 reported that paroxetine daily improved IELT and the ability to control ejaculation more effectively than behaviour therapy, but it improved sexual satisfaction less [32]. Wang et al. reported that 18.3 and 36.7% of patients in the paroxetine and behaviour therapy groups, respectively, withdrew from the study due to lack of efficacy or adverse effects [15]. The rates of occurrence of side effects were 24.4 and 1.6% in the paroxetine and behaviour therapy groups [15, 30, 32], respectively. No significant differences were observed between the two groups [RR, 8.66; 95% Cl, 0.83 to 90.11; *p* = 0.07] [15, 30, 32] (Fig. 4).

Paroxetine vs. local lidocaine gel: A single RCT [20] reported that paroxetine-treated patients had a longer IELT, of 3.25 min than had those treated with lidocaine gel [MD, 0.13; 95% [Cl], − 0.58 to 0.84,*p* = 0.72] (Fig. 3), and paroxetine was associated with better sexual satisfaction scores than the local anaesthetic of 3.25 and 2.97 points, respectively. The most common side effects were penile anaesthesia and headache in the lidocaine and paroxetine groups, respectively.

Paroxetine vs. duloxetine: A single RCT [33] provided evidence suggesting that the difference in IELT was not significant between the two groups at 1 month of treatment [MD, 0.05; 95% [Cl], − 0.01 to 0.11;*p* = 0.1] (Fig. 3). Drug-related side effects included yawning and somnolence (25%), nausea (25%) and asthenia (10%) in the paroxetine group and nausea (25%), headache (12.5%), dry mouth (2.5%), constipation (5%) and dizziness (7.5%) in the duloxetine group.

Paroxetine vs. escitalopram: Only one RCT [31] compared IELT and adverse events between paroxetine and escitalopram groups. Treatment with paroxetine was found to be significantly more effective based on IELT than escitalopram [MD, 0.2; 95% [Cl], 0.08 to 0.32;*p* = 0.001] (Fig. 3). Both drugs were generally well tolerated.

**Fig. 5:**
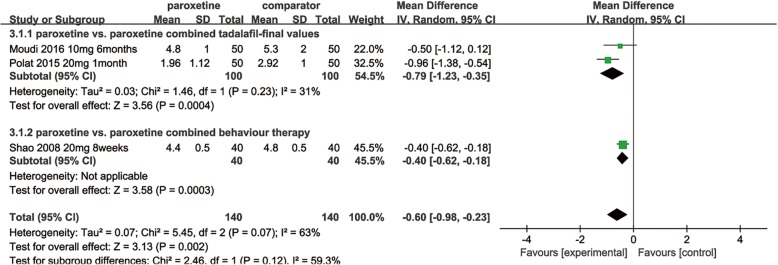
Pooled estimate of IELT of paroxetine vs. combined therapy

Paroxetine combined with tadalafil vs. paroxetine: The between-group difference in IELT, based on 2 RCTs [16, 26] comparing daily paroxetine alone with paroxetine combined with tadalafil, was − 0.79 in favour of the latter [95% Cl, − 1.23 to − 0.35;*p* = 0.0004] (Fig. 5), while there was no significant difference in side effects [RR, 0.80; 95% [Cl], 0.34 to 1.88; *p* = 0.6] [16, 26] (Fig. 4).

Paroxetine combined with behaviour therapy vs. paroxetine: A single RCT [32] reported that patients treated with paroxetine combined with behaviour therapy had a significantly longer IELT than had those treated with paroxetine alone [MD, − 0.40; 95% [Cl], − 0.62 to − 0.18;*p* = 0.0003] (Fig. 5). The rates of occurrence of side effects were 10.0 and 40% in the paroxetine and combined groups, respectively, but all side effects, including dizziness, dry mouth, sleep disturbances and fatigue, were tolerable.

## Discussion

This meta-analysis, including 19 RCTs comparing the efficacy and safety of paroxetine with other drugs for PE, showed that, compared with placebo, fluoxetine and escitalopram, paroxetine could improve IELT significantly with tolerable side effects. However, when comparing paroxetine with tramadol, sertraline, PDE5Is, local lidocaine gel, behaviour therapy or dapoxetine, we found that the increase in IELT was not statistically significant. Furthermore, paroxetine combined with tadalafil or behaviour therapy was more efficacious than paroxetine alone (all *p* < 0.05). According to published articles from 1998 to 2018, the definition of PE has changed greatly, and the main outcomes only include IELT. Other diffused distribution outcomes included sexual satisfaction score, side effects, PEDT, IIEF, PEP, AIPE, libido and frequency of intercourse, causing difficulty in pooling for meta-analysis. Based on the new ISSM guidelines [11], PE is more than only a short time to ejaculation, and the ability to control ejaculation and personal emotions have been identified as important factors. Therefore, it is not convincing to us to compare IELT only between different drugs in the treatment of PE. Analysing the diffused distribution outcomes, some RCTs reported that paroxetine was associated with better sexual satisfaction than placebo, dapoxetine and lidocaine gel [18, 20, 22, 23], better PEP than placebo and tramadol [17], a better ejaculation-delay effect than placebo and behaviour therapy [21, 22, 32], better mean numbers of coitus episodes than placebo and dapoxetine [22], a better PEDT and IIEF values than placebo and better libido and AIEF than tramadol [21, 23, 25]. Conversely, evidence from other RCTs showed that there was no significant difference in sexual satisfaction between paroxetine and tramadol or dapoxetine [20, 23], no difference in PEDT between paroxetine and PDE5Is [23], and no difference in AIEF between paroxetine and fluoxetine [31]. The possible reasons for these results include the different types of PE and treatment periods.

The ejaculation reflex mainly includes two pathways: peripheral and central. First, during intercourse, the glans of the penis is stimulated, triggering the pudendal sensory nerves; the signal is then transferred to the spinal cord. After the spinal cord receives the signal, the sensory information is converted into secretory and motor signal output, which induces contraction of the epididymis, vas deferens, seminal vesicles, prostate and bladder neck, in turn rhythmically leading to ejaculation through the distal urethra [34]. The ejaculation reflex is also associated with serotonin and dopamine in the central nervous system. The most researched neurotransmitter is 5-HT. Some subtypes (5-HT1a) reduce ejaculatory latency, and other subtypes (5-HT1b, 5-HT2c) prolong ejaculatory latency [35–37]. Paroxetine is one of the SSRIs that increases the amounts of 5-HT in postsynaptic membrane receptors and thus delays ejaculation. Although not approved by the FDA, compared with the dapoxetine, it has the advantage of lower dropouts and cost with almost identical effects [12, 13]. According to a single RCT [22], paroxetine increased IELT significantly more than dapoxetine (*p* = 0.01). Furthermore, weekly intercourse frequency and satisfaction score were also improved, and the side effects were well tolerated [22, 23]. The difference in IELT between paroxetine and dapoxetine requires more RCTs to confirm.

We evaluated the heterogeneity of each comparison regarding the pooled results by excluding single studies sequentially. The results showed that the heterogeneity remained at a high level in the paroxetine vs. placebo group and the paroxetine vs. tramadol group, validating the rationality of our outcomes. The reasons for this finding might have arisen from the different types of PE, treatment periods and sample sizes. However, in the paroxetine vs. PDE5Is and paroxetine vs. fluoxetine groups, when excluded Polat’s and Otunctemur’s articles, the results showed that the heterogeneity decreased from 88 and 77% to 46 and 0%, respectively. Analysing the data from Polat’s study, a similar IELT value following treatment with paroxetine or PDE5Is might be the cause of this situation. However, Based on data from Otunctemur’s study, the heterogeneity might have been due to several design differences among the studies,including patient selection and limited sample sizes. With this point in mind, we excluded their research, and the results of the sensitivity analysis showed that the between-group difference in IELT, based on 2 RCTs [15, 20] comparing daily paroxetine with PDE5Is, was − 0.98 in favour of PDE5Is [95% Cl, − 1.55 to − 0.41; *p* = 0.0007]. Further, based on 3 pooled RCTs [19, 28, 31], the patients treated with paroxetine 20 mg had significantly increased IELT, compared with fluoxetine (*p* < 0.0001). The MD in IELT was 0.29 in favour of paroxetine [(random effect) 95% [Cl], 0.15 to 0.43; *p* < 0.0001]. The between-group difference in side effects, after excluding 1 RCT [24] comparing daily paroxetine with placebo, was 2.32, indicating that paroxetine has more side effects than placebo [RR, 2.32; 95% Cl, 1.15 to 4.68;*p* = 0.02]. The heterogeneity decreased from 65 to 0%. It is believed that the results from Abdel-Hamidi et al. could become the basis of heterogeneity. A similar outcome was detected (I^2^ 67 to 0%) when excluding Zhang’s report [30], indicating that paroxetine has more side effects than behaviour therapy [RR, 26.93; 95% [Cl], 3.76 to 192.88; *p* = 0.001], although we should be cautious about these results because of limited pooled RCTs and the clinical heterogeneity of the recruited participants, along with the lack of clarify regarding the methodological quality.

To the best of our knowledge, this study is the first meta-analysis to report the efficacy and safety of paroxetine in the treatment of PE. Therefore, it is necessary for urologists to update these articles because paroxetine is still not approved by the FDA. Furthermore, the incidence of drug-related side effects, such as dizziness, dry mouth, sleep disturbances and fatigue, could be pooled and evaluated between different drugs.

There were some limitations to this meta-analysis. First, the definition of PE is controversial. The new characteristics of PE are described above [11]. While more than 8 types of definition could be seen in 19 RCTs, mainly IELT, some RCTs defined PE as IELT< 2 min, < 1 min, and < 1.5 min, and some articles used the DSM-IV guideline, which might have influenced the results in the end. For example, if patient A improved primarily in IELT from 25 s to 59 s (PE defined as IELT < 1 min), and patient B improved in IELT from 119 s to 125 s (PE defined as IELT < 2 min) after treatment, it indicated that patient A had better efficacy in light of the change from baseline. However, an increase in IELT from 25 to 59 s (still < 1 min) was likely not clinically relevant based on the definition of PE, and we could only compare the end values, such as 59 and 125, regarding IELT. Therefore, the difference between the two groups could have caused false positives. It is recommended that subgroup analysis be performed according to the same definition of PE in the future. Second, the majority of the included RCTs were 4–12 weeks in duration. Only Wang’s and Moudi’s trials lasted 6 months [15, 16]. A longer-term follow-up should be performed to explore the efficacy and safety of paroxetine. Third, we searched PubMed, Embase and Cochrane Central Register but excluded quasi-randomized trials, non-randomized trials, observational studies, case reports, abstract and letters, which resulted in a sample that was not large. Fourth, funnel plots could not be used to assess publication bias because of insufficient RCT comparisons. Finally, only one RCT compared local lidocaine, duloxetine and escitalopram with paroxetine in meta-analysis, which could have increased the likelihood of false negatives and false positives. In all, more high-quality RCTs should be performed to address the efficacy and safety of these drugs in the treatment of PE.

## Conclusions

Paroxetine has the advantage of improving IELT with well-tolerated side effects, compared with placebo, fluoxetine and escitalopram. Although only dapoxetine has been approved by the FDA, it does not mean that dapoxetine was more effective than other drugs. According to this meta-analysis and systematic review, paroxetine has good efficacy in the treatment of PE.

Future RCTs should also be unified with the definition of PE and evaluate sexual satisfaction, IIEF, PEP, PEDT, etc. A longer-term follow-up should be performed to explore the efficacy and safety of paroxetine.

## Abbreviations

SSRIs: Selective serotonin reuptake inhibitors
PE: Premature ejaculation
IELT: Intra-vaginal ejaculatory latency time
RCTs: Randomized controlled trials
PDE5Is: Phosphodiesterase 5 inhibitors
ISSM: International Society for Sexual Medicine
FDA: Food and Drug Administration
5-HT: 5-hydroxytryptamine
MD: Mean difference
RR: Relative risk
PEDT: Premature ejaculation diagnostic tool
IIEF: International Index of Erectile Function
PEP: Premature ejaculation profile
AIPE: Arabic index of Premature Ejaculation
CIPE-5: Chinese index of premature ejaculation
PEDT: Premature ejaculation diagnostic tool
PE: Premature ejaculation
SD: Standard deviation
DSM-IV: Diagnostic and statistical manual of mental disorders, fourth edition
Cl: Confidence interval

## References

[1] Porst H, Montorsi F, Rosen R C, Gaynor L,Grupe S, Alexander J. The premature ejaculation prevalence and attitudes (PEPA) survey: prevalence, comorbidities, and professional help-seeking [J] Eur Urol, 2007,51(3):816–823, 824. DOI:10.1016/j.eururo.2006.07.004. PMID:16934919 10.1016/j.eururo.2006.07.004 16934919

[2] Kempeneers P, Andrianne R, Cuddy M, Blairy S. Sexual cognitions, trait anxiety, sexual anxiety, and distress in men with different subtypes of premature ejaculation and in their partners [J]. J Sex Marital Ther, 2018,44(4):319–332. DOI:10.1080/0092623X.2017.1405299 .PMID:29161211. 10.1080/0092623X.2017.1405299 29161211

[3] Saitz T R, Serefoglu E C. Advances in understanding and treating premature ejaculation [J]. Nat Rev Urol, 2015,12(11):629–640. DOI:10.1038/nrurol.2015.252 .PMID:26502991. 10.1038/nrurol.2015.252 26502991

[4] Safarinejad MR (2015). Retraction statement: analysis of association between the 5-HTTLPR and STin2 polymorphisms in the serotonin-transporter gene and clinical response to a selective serotonin reuptake inhibitor (sertraline) in patients with premature ejaculation [J]. BJU Int.

[5] Jern P, Eriksson E, Westberg L (2013). A reassessment of the possible effects of the serotonin transporter gene linked polymorphism 5-HTTLPR on premature ejaculation [J]. Arch Sex Behav.

[6] Mourikis I, Antoniou M, Matsouka E, Vousoura E, Tzavara C, Ekizoglou C (2015). Anxiety and depression among Greek men with primary erectile dysfunction and premature ejaculation [J]. Ann General Psychiatry.

[7] Brody S, Weiss P (2015). Erectile dysfunction and premature ejaculation: interrelationships and psychosexual factors [J]. J Sex Med.

[8] Salama N, Eid A, Swedan A, Hatem A (2017). Increased prevalence of premature ejaculation in men with metabolic syndrome [J]. Aging Male.

[9] Lee JH, Lee SW (2015). Relationship between premature ejaculation and chronic prostatitis/chronic pelvic pain syndrome [J]. J Sex Med.

[10] Sansone A, Romanelli F, Jannini EA, Lenzi A (2015). Hormonal correlations of premature ejaculation [J]. Endocrine.

[11] Serefoglu EC, McMahon CG, Waldinger MD, Althof SE, Shindel A, Adaikan G (2014). An evidence-based unified definition of lifelong and acquired premature ejaculation: report of the second international society for sexual medicine ad hoc committee for the definition of premature ejaculation [J]. Sex Med.

[12] Jern P, Johansson A, Piha J, Westberg L, Santtila P (2015). Antidepressant treatment of premature ejaculation: discontinuation rates and prevalence of side effects for dapoxetine and paroxetine in a naturalistic setting [J]. Int J Impot Res.

[13] Simsek A, Kirecci SL, Kucuktopcu O, Ozgor F, Akbulut MF, Sarilar O (2014). Comparison of paroxetine and dapoxetine, a novel selective serotonin reuptake inhibitor in the treatment of premature ejaculation [J]. Asian J Androl.

[14] Higgins JP, Altman DG, Gotzsche PC, Jüni P, Moher D, Oxman AD (2011). The Cochrane Collaboration's tool for assessing risk of bias in randomised trials [J]. BMJ.

[15] Wang WF, Wang Y, Minhas S, Ralph DJ (2007). Can sildenafil treat primary premature ejaculation? A prospective clinical study [J]. Int J Urol.

[16] Moudi E, Kasaeeyan AA (2016). Comparison between Tadalafil plus paroxetine and paroxetine alone in the treatment of premature ejaculation [J]. Nephrourol Mon.

[17] Hamidi-Madani A, Motiee R, Mokhtari G, Nasseh H, Esmaeili S, Kazemnezhad E (2018). The efficacy and safety of on-demand tramadol and paroxetine use in treatment of life long premature ejaculation: a randomized double-blind placebo-controlled clinical trial [J]. J Reprod Infertil.

[18] Gong ZY, Tang TL, Cui S, Wang JZ, Deng XZ (2011). [Oral paroxetine for premature ejaculation: a randomized controlled study][J]. Zhonghua Nan Ke Xue.

[19] Waldinger MD, Hengeveld MW, Zwinderman AH, Olivier B (1998). Effect of SSRI antidepressants on ejaculation: a double-blind, randomized, placebo-controlled study with fluoxetine, fluvoxamine, paroxetine, and sertraline [J]. J Clin Psychopharmacol.

[20] Gameel TA, Tawfik AM, Abou-Farha MO, Bastawisy MG, El-Bendary MA, Ael-N E-G (2013). on-demand use of tramadol, sildenafil, paroxetine and local anaesthetics for the management of premature ejaculation: a randomised placebo-controlled clinical trial [J]. Arab J Urol.

[21] Sunay D, Sunay M, Aydogmus Y, Bağbancı S, Arslan H, Karabulut A (2011). Acupuncture versus paroxetine for the treatment of premature ejaculation: a randomized, placebo-controlled clinical trial [J]. Eur Urol.

[22] Safarinejad MR (2006). Comparison of dapoxetine versus paroxetine in patients with premature ejaculation: a double-blind, placebo-controlled, fixed-dose, randomized study [J]. Clin Neuropharmacol.

[23] Abu E M, Abdelhamed A. Comparison of the clinical efficacy and safety of the on-demand use of paroxetine, dapoxetine, sildenafil and combined dapoxetine with sildenafil in treatment of patients with premature ejaculation: A randomised placebo-controlled clinical trial [J]. Andrologia, 2018,50(1). DOI:10.1111/and.12829. PMID:28497478 10.1111/and.12829 28497478

[24] Abdel-Hamid IA, El NE, El GA (2001). Assessment of as needed use of pharmacotherapy and the pause-squeeze technique in premature ejaculation [J]. Int J Impot Res.

[25] Alghobary M, El-Bayoumy Y, Mostafa Y, Mahmoud el-HM,Amr M. Evaluation of tramadol on demand vs. daily paroxetine as a long-term treatment of lifelong premature ejaculation [J]. J Sex Med, 2010,7(8):2860–2867. DOI:10.1111/j.1743-6109.2010.01789.x. PMID:20367773 10.1111/j.1743-6109.2010.01789.x 20367773

[26] Polat EC, Ozbek E, Otunctemur A, Ozcan L, Simsek A (2015). Combination therapy with selective serotonin reuptake inhibitors and phosphodiesterase-5 inhibitors in the treatment of premature ejaculation [J]. Andrologia.

[27] Otunctemur A, Ozbek E, Kirecci SL, Ozcan L, Dursun M, Cekmen M (2014). Relevance of serum nitric oxide levels and the efficacy of selective serotonin reuptake inhibitors treatment on premature ejaculation: decreased nitric oxide is associated with premature ejaculation [J]. Andrologia.

[28] Kirecci SL, Simsek A, Gurbuz ZG, Mimaroglu S, Yuksel A, Vural P (2014). Relationship between plasma melatonin levels and the efficacy of selective serotonin reuptake inhibitors treatment on premature ejaculation [J]. Int J Urol.

[29] Kirecci SL, Simsek A, Gurbuz ZG, Gurdal H, Gurbuz ZG, Usanmaz S (2014). "relevance of seminal plasma nitric oxide levels and the efficacy of SSRI treatment on lifelong premature ejaculation." [J]. Andrologia.

[30] Zhang WX, Qin JC, Wang R, Lei W, Jie Z (2012). Effect evaluation of paroxetine and sertraline in the treatment of premature ejaculation [J]. Chinese journal of andrology.

[31] Arafa M, Shamloul R (2007). A randomized study examining the effect of 3 SSRI on premature ejaculation using a validated questionnaire [J]. Ther Clin Risk Manag.

[32] Shao XY, Li JB (2008). Clinical study on treatment of premature ejaculation with paroxetine and behavior-therapy [J]. Chinese journal of andrology.

[33] Ozcan L, Polat EC, Otunctemur A, Ozbek E (2015). Duloxetine, dual serotonin and norepinephrine reuptake inhibitor, versus paroxetine, selective serotonin reuptake inhibitor, in the treatment for premature ejaculation [J]. Int Urol Nephrol.

[34] Puppo V, Puppo G (2016). Comprehensive review of the anatomy and physiology of male ejaculation: premature ejaculation is not a disease [J]. Clin Anat.

[35] Giuliano F, Clément P (2006). Serotonin and premature ejaculation: from physiology to patient management. [J]. Eur Urol.

[36] Jannuni EA, Carosa E, Pepe M, Lombardo F, Lenzi A (2006). Update on pathophysiology of premature ejaculation: the bases for new pharmacological treatments. [J]. EAU-EBU Update Series.

[37] Janssen PK, van Schaik R, Zwinderman AH, Olivier B, Waldinger MD (2014). The 5-HT(1) a receptor Caucasian men with lifelong premature ejaculation [J]. Pharmacol Biochem Behav.

